# Supplementary material for: Caulis Polygoni Multiflori Accelerates Megakaryopoiesis and Thrombopoiesis via Activating PI3K/Akt and MEK/ERK Signaling Pathways
Source: Pharmaceuticals (Basel). 2022 Sep 28;15(10):1204. doi: 10.3390/ph15101204 (PMC9607024; doi:10.3390/ph15101204)
Supplement: Supplementary file 1 [file pharmaceuticals-15-01204-s001.zip › Supplementary Figures.pdf]

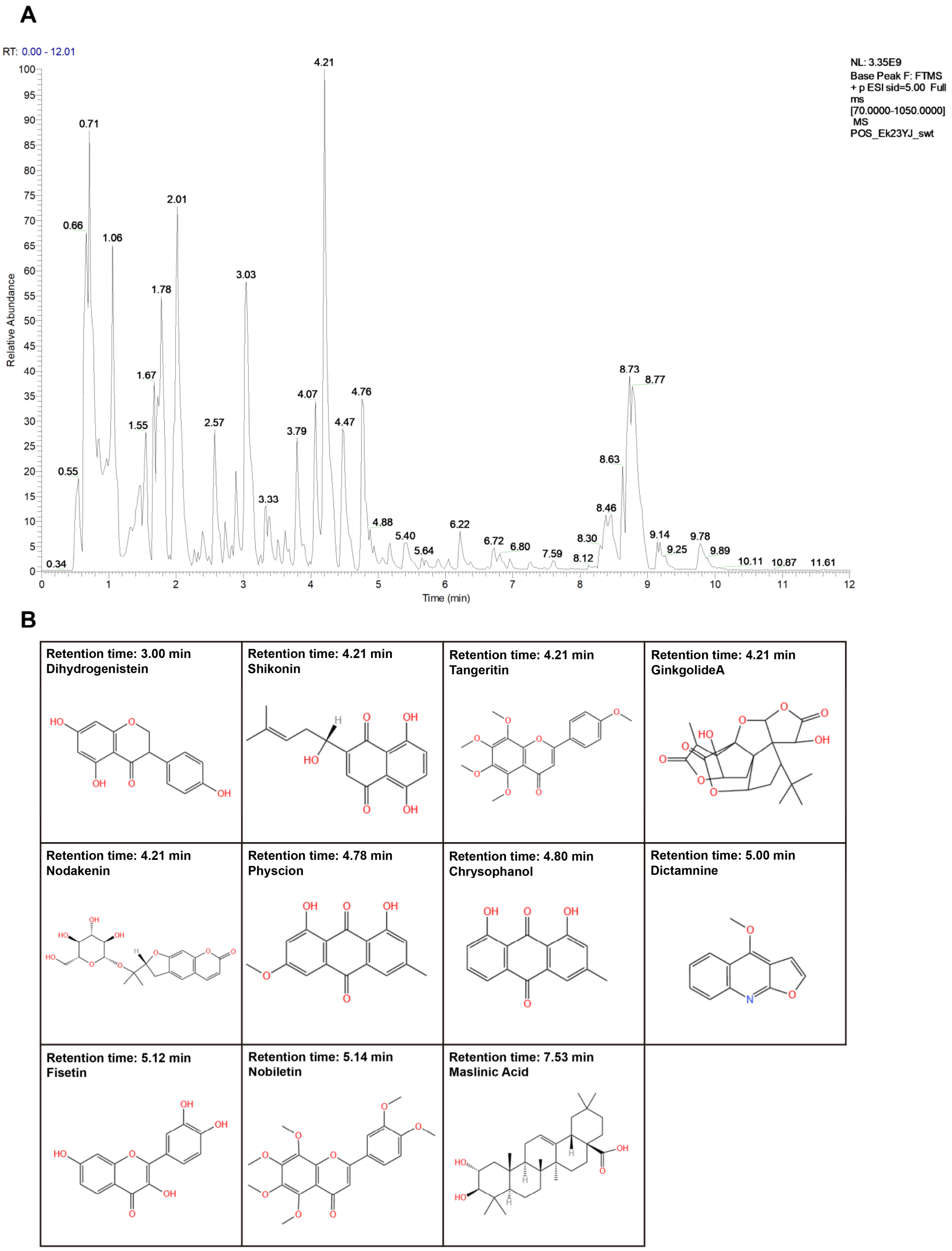


**Figure S1.** Characterization of CPM by UHPLC QE HF-X MS. (**A**) Total ion chromatogram of CPM. (**B**) Retention times, names and chemical structures of these 11 characterized compounds.

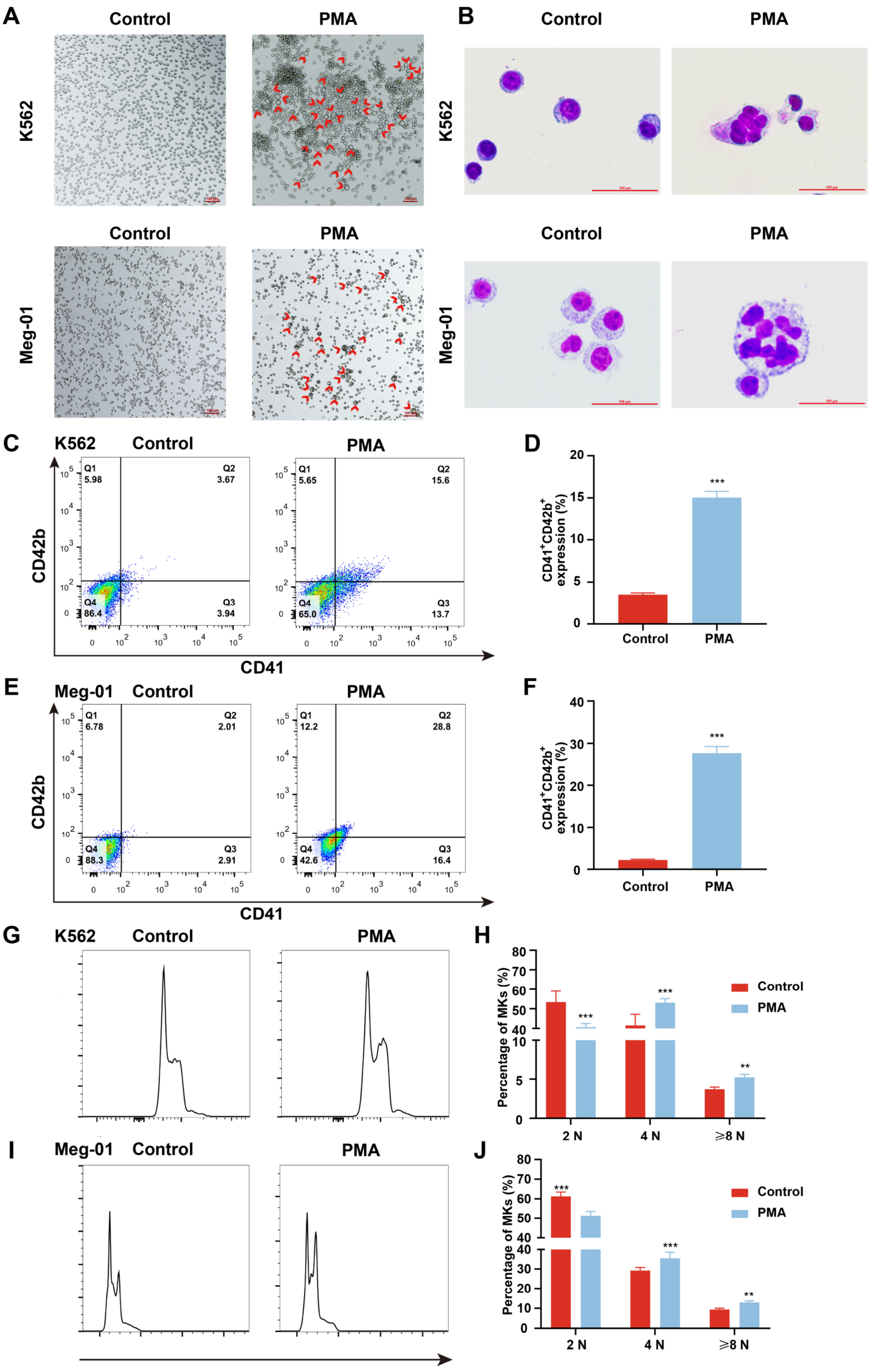


**Figure S2.** MK differentiation of K562 and Meg-01 cells induced by PMA. (**A**) Microscope photographs of K562 and Meg-01 cells with or without PMA treatment (1 nM) for 5 days were randomly captured at 10× resolution under the inverted light microscope. Scar bar: 100 μm. (**B**)

Giemsa staining of K562 and Meg-01 cells treated with or without PMA (1 nM) for 5 days. Scar bar: 100 μm. (**C**, **E**) CD41 and CD42b expression of K562 and Meg-01 cells with or without PMA (1 nM) treatment for 5 days. (**D**, **F**) The proportion of $CD41^{+}CD42b^{+}$ cells in control and PMA-treated groups. Data are mean ± SD (n=3). (**G**, **I**) DNA ploidy analysis of K562 and Meg-01 cells with or without PMA (1 nM) treatment for 5 days. (**H**, **J**) The percentage of 2N, 4N and ⩾ 8N cells in control and PMA-treated group. Data are mean ± SD (n=3, ANOVA). * $p < 0.05$, ** $p < 0.01$, *** $p < 0.001$ vs. the control group.
